# Identification and Characterization of EvpQ, a Novel T6SS Effector Encoded on a Mobile Genetic Element in *Edwardsiella piscicida*

**DOI:** 10.3389/fmicb.2021.643498

**Published:** 2021-03-11

**Authors:** Duan You Li, Ying Li Liu, Xiao Jian Liao, Tian Tian He, Shan Shan Sun, Pin Nie, Hai Xia Xie

**Affiliations:** ^1^State Key Laboratory of Freshwater Ecology and Biotechnology, Key Laboratory of Aquaculture Disease Control, Ministry of Agriculture, Institute of Hydrobiology, Chinese Academy of Sciences, Wuhan, China; ^2^College of Advanced Agricultural Sciences, University of Chinese Academy of Sciences, Beijing, China; ^3^Laboratory for Marine Biology and Biotechnology, Pilot National Laboratory for Marine Science and Technology, Qingdao, China; ^4^School of Marine Science and Engineering, Qingdao Agricultural University, Qingdao, China

**Keywords:** type VI secretion system, effector, EvpQ, regulation, mobile genetic element, *Edwardsiella piscicida*

## Abstract

In this study, a hypothetical protein (ORF02740) secreted by *Edwardsiella piscicida* was identified. We renamed the ORF02740 protein as EvpQ, which is encoded by a mobile genetic element (MGE) in *E. piscicida* genome. The *evpQ* gene is spaced by 513 genes from type VI secretion system (T6SS) gene cluster. Low GC content, three tRNA, and three transposase genes nearby *evpQ* define this MGE that *evpQ* localizes as a genomic island. Sequence analysis reveals that EvpQ shares a conserved domain of C70 family cysteine protease and shares 23.91% identity with T3SS effector AvrRpt2 of phytopathogenic *Erwinia amylovora.* Instead, EvpQ of *E. piscicida* is proved to be secreted at a T6SS-dependent manner, and it can be translocated into host cells. EvpQ is thereof a novel T6SS effector. Significantly decreased competitive index of Δ*evpQ* strain in blue gourami fish (0.53 ± 0.27 in head kidney and 0.44 ± 0.19 in spleen) indicates that EvpQ contributes to the pathogenesis of *E. piscicida*. At 8-, 18-, and 24-h post-subculture into DMEM, the transcription of *evpQ* was found to be negatively regulated by Fur and positively regulated by EsrC, and the steady-state protein levels of EvpQ are negatively controlled by RpoS. Our study lays a foundation for further understanding the pathogenic role of T6SS in edwardsiellosis.

## Introduction

*Edwardsiella piscicida*, previously known as *Edwardsiella tarda*, is a Gram-negative intracellular pathogen (Abayneh et al., 2013; Shao et al., 2015). It causes edwardsiellosis in more than 20 important species of farmed fish, especially flounder (*Paralichthys olivaceus*) and turbot (*Scophthalmus maximus*) (Matsuyama et al., 2005; Padrós et al., 2006; Mohanty and Sahoo, 2007). The survival and replication of *E. piscicida* in its hosts depends on its type VI secretion system (T6SS) and type III secretion system (T3SS) (Tan et al., 2005; Okuda et al., 2006; Zheng and Leung, 2007).

Srinivasa Rao et al. (2004) identified the *Edwardsiella* virulent proteins (EVPs) from *E. piscicida* PPD130/91 in a mass spectrometric screen for secreted virulence factors. These EVPs were analogous to the T6SS proteins named first by Pukatzki et al. (2006). *E. piscicida* T6SS are encoded in a gene cluster which contains 13 conserved core genes encoding proteins necessary to assemble the basic secretion apparatus (Zheng and Leung, 2007). Besides the core apparatus protein, EvpP (T6SS effector protein), EvpC (a homolog of Hcp), and EvpI (a homolog of VgrG) are three secreted proteins encoded inside the T6SS gene cluster (Zheng and Leung, 2007). T6SS is a contractile injection apparatus that translocates a tail tube and a spike loaded with various effectors directly into eukaryotic or prokaryotic target cells (Silverman et al., 2013; Flaugnatti et al., 2016; Hernandez et al., 2020; Wettstadt and Filloux, 2020). The T6SS spike punctures the bacterial cell envelope allowing the transport of effectors, and it consists of a torch-like VgrG trimer (encoded by *evpI* in *E. piscicida*) on which sits a PAAR protein sharpening the VgrG tip (encoded by *evpJ* in *E. piscicida*), and VgrG itself sits on the Hcp tube (encoded by *evpC* in *E. piscicida*). All these elements are packed into a T6SS sheath and are propelled out of the bacterial cell and into target cells. *Vibrio cholerae* VgrG-1 is the first identified T6SS effector, which is secreted by T6SS and displays a C-terminal actin cross-linking domain upon its translocation into phagocytic target cells (Pukatzki et al., 2007; Ma et al., 2009). EvpP is the first T6SS effector identified in *Edwardsiella* spp., and it can target intracellular Ca^2+^ signaling to impair Jnk activation and ASC oligomerization, therefore promoting bacterial colonization (Chen et al., 2017).

*Edwardsiella piscicida* T6SS plays a pivotal role at the early infection stage *in vivo* when T3SS expression is repressed by alternative sigma factor RpoS (Yang et al., 2017; Yin et al., 2018). The two-component system sensor PhoQ detects changes of environmental temperature as well as Mg^2+^ concentration, and the response regulator PhoP regulates the T6SS through direct activation of *esrB* in *E. piscicida* (Chakraborty et al., 2010). PhoR-PhoB two-component system senses changes in Pi concentration, and the ferric uptake regulator (Fur) senses changes in iron concentration to regulate the expression of T6SS (Chakraborty et al., 2011). PhoB and EsrC bind simultaneously on DNA upstream of *evpA*, tightly regulating the transcription of whole T6SS cluster, except the T6SS effector gene *evpP*. Fur senses high iron concentration and binds directly to the Fur box in the promoter of *evpP* to inhibit the binding of EsrC to the same region (Chakraborty et al., 2011). The elegantly regulated T6SS contributes to the LD_50_ of *E. piscicida* by ∼100 times (Zheng and Leung, 2007).

In this study, the novel T6SS effector, EvpQ, was identified in *E. piscicida*. EvpQ was revealed to be encoded on a genomic island, and its regulation network and contribution to virulence was characterized.

## Materials and Methods

### Bacterial Strains

*Edwardsiella tarda* PPD130/91 was recognized as *Edwardsiella piscicida* PPD130/91 ever since 2015 (Ling et al., 2000; Shao et al., 2015). The *E. piscicida* strains and plasmids used in this study are described in Table 1. *E. piscicida* strains were grown in TSB medium static. To activate T6SS and T3SS, *E. piscicida* and its derived strains were grown in Dulbecco’s modified Eagle’s medium (DMEM, Invitrogen) at 25°C under 5% (*v*/*v*) CO_2_ atmosphere. When required, appropriate antibiotics were supplemented at the concentrations of 100 μg/ml ampicillin (Amp; Sigma), 12.5 μg/ml colistin (Col; Sigma), 34 μg/ml chloramphenicol (Cm; Amresco), 15 μg/ml tetracycline (Tet; Amresco), and 50 μg/ml kanamycin (Km; BioFroxx).

**TABLE 1:** Strains and plasmids used in this study.

Strain or plasmid	Description and/or genotype	Reference or source
**Bacteria**		
***Edwardsiella piscicida***		
PPD130/91	Wild type, Km^*s*^, Col^*r*^, Amp^*s*^, and LD_50_ = 10^5.0^	Ling et al. (2000)
WT *evpQ*:2HA	PPD130/91with chromosomal expression of *evpQ*-2HA, Amp^*r*^, and Km^*r*^	This study
Δ*esaB*	PPD130/91, *esaB* in-frame deletion of aa 1 to 149	Liu et al. (2017)
Δ*esaB*Δ*esaN*	PPD130/91, in-frame deletion of *esaB* and *esaN*	This study
Δ*esaN*	PPD130/91 with in-frame deletion of *esaN*	Xie et al. (2010)
Δ*esaN evpQ*::2HA	Δ*esaN* with chromosomal expression of *evpQ*-2HA, Amp^*r*^, and Km^*r*^	This study
Δ*evpO*	PPD130/91, *evpO* in-frame deletion of aa 13 to 1257	Zheng and Leung (2007)
Δ*evpO evpQ*::2HA	Δ*evpO* with chromosomal expression of *evpQ*-2HA, Amp^*r*^, Km^*r*^	This study
Δ*esaB evpQ*::2HA	Δ*esaB* with chromosomal expression of *evpQ*-2HA, Amp^*r*^, and Km^*r*^	This study
Δ*evpQ*	PPD130/91, ETAE_2037 in-frame deletion of aa 1 to 173	This study
Δ*evpQ*::km	PPD130/91, ETAE_2037 replaced with Km^*r*^	This study
Δ*rpoS*	PPD130/91, *rpoS* in-frame deletion of aa 1 to 490	This study
Δ*rpoS evpQ*::2HA	Δ*rpoS* with chromosomal expression of *evpQ*-2HA, Amp^*r*^, and Km^*r*^	This study
Δ*esrC*	PPD130/91, *esrC* in-frame deletion of aa 12 to 220	Zheng et al. (2005)
Δ*fur*	PPD130/91 with in-frame deletion of *fur* protein aa 1–112	Zhang et al. (2019)
Δ*fur evpQ*::2HA	Δ*fur* with chromosomal expression of *evpQ*-2HA, Amp^*r*^, and Km^*r*^	This study
WT/pACYC-*eseG*::*cyaA*	PPD130/91 with pACYC-*eseG::cyaA*	Lu et al. (2016)
Δ*esaN*/pACYC-*eseG*::*cyaA*	Δ*esaN* with pACYC-*eseG*::*cyaA*	Lu et al. (2016)
WT/pACYC-*evpQ*::*cyaA*	PPD130/91 with pACYC-*evpQ::cyaA*	This study
Δ*esaN*/pACYC-*evpQ*::*cyaA*	Δ*esaN* with pACYC-*evpQ*::*cyaA*	This study
Δ*evpO*/pACYC-*evpQ*::*cyaA*	Δ*evpO* with pACYC-*evpQ*::*cyaA*	This study
WTpFPV-P-*evpQ*_–__594 to__–__1_	PPD130/91 with pFPV-P-*evpQ*_–__594*to*__–__1_	This study
Δ*esrC/*pFPV-P-*evpQ*_–__594 to__–__1_	Δ*esrC* with pFPV-P-*evpQ*_–__594 to__–__1_	This study
Δ*rpoS/*pFPV-P-*evpQ*_–__594 to__–__1_	Δ*rpoS* with pFPV-P-*evpQ*_–__594 to__–__1_	This study
Δ*fur/*pFPV-P-*evpQ*_–__594 to__–__1_	Δ*fur* with pFPV-P-*evpQ*_–__594 to__–__1_	This study
***Escherichia coli***		
DH5α	α Complementation	Stratagene
S17-1 λpir	RK2 *tra* regulon, λ*pir*	Simon et al. (1983)
**Plasmids**		
pMD18-T	Cloning vector, Amp^*r*^	TaKaRa
pRE112	Suicide plasmid, *pir*dependent, Cm^*r*^, *oriT*, *oriV*, and *sacB*	Edwards et al. (1998)
pACYC-184	Tet^*r*^ and Cm^*r*^	Amersham
pACYC-*evpQ*-HA	Tet^*r*^	This study
pKD46	Red helper plasmid, Amp^*r*^	Datsenko and Wanner (2000)
pKD4	Template plasmid for PCR amplify FRT-flanked resistance gene, Km^*r*^ and Amp^*r*^	Datsenko and Wanner (2000)
pSU315	Template plasmid with FLP recognition target site and 2HA tag sequence, Amp^*r*^, and Km^*r*^	Uzzau et al. (2001)
pFPV25	Plasmid with promoterless *gfp* gene	Valdivia and Falkow (1996)
pFPV-P-*evpQ*_–__594 to__–__1_	-594 to -1 of *evpQ* inserted into upstream of *gfp* in pFPV25	This study

**Col*, colistin; *Amp*, ampicillin; *Tet*, tetracycline; *Cm*, chloramphenicol; Km, kanamycin; Superscripts: *r*, resistance; *s*, sensitivity.*

### Cell Line and Cultivation

Epithelioma papillosum of carp (*Cyprinus carpio*) (EPC) cells (Wolf and Mann, 1980) were grown in M199 medium (HyClone), supplemented with 10% fetal bovine serum (FBS, Gibco). EPC cells were maintained in a 5% CO_2_ atmosphere at 25°C.

### Mutant Strain Construction

Using the λ Red recombination system as described previously (Datsenko and Wanner, 2000), the chromosomal copy of *evpQ* gene was replaced with kanamycin gene, obtaining the strain Δ*evpQ*::km. The scarless mutant strains Δ*evpQ* and Δ*rpoS* were obtained by *sacB*-based allelic exchange using a method as previously described (Edwards et al., 1998; Zheng et al., 2005). All mutants obtained were verified by DNA sequencing. All primers used are listed in Table 2.

**TABLE 2:** Oligonucleotides used in this study.

Designation	Nucleotide sequence
*evpQ*-for	GCTCTAGAACCCAGCAGCCTGACATTG
*evpQ*-int-rev	TTTCGGCCTCACCTCTGATAGTTA
*evpQ*-int-for	ATCAGAGGTGAGGCCGAAAATG GCTATTTTTGCTCAGCTC
*evpQ*-rev	GCTCTAGA TCTTTAACGGTTGGGGATG
*evpQ*-com-for	GGAATTCATTGGGGAGGGGTAGAGTG
*evpQ*-com-rev	AAAAGTACTTTAAGCGTAATCT GGAACATCGTATGGGTAATT TGGCTCAAGCAATGGT
*rpoS*-for	ATGGTACCTACGCTGGTTACAATGTGGCT
*rpoS*-int-rev	AGCTGTACCCTACCCGTGATTTG
*rpoS*-int-for	CAAATCACGGGTAGGGTACAGC TGCGATGCGGTCAAAAAAAACGG
*rpoS*-rev	ATGGTACCGCTACGTCCGCCCACAGCTGA
pSU315-*evpQ::*2HA for	CATTGACATAAATGTGCTGACACGG AGCAAAGGGAGGCGGTCCATA TGAATATCCTCCTTAGT
pSU315-*evpQ-*rev	TATAGGCTATCGTTAACCAG GCAAGGATTCCATGTATCA TCACATATGAATATCCTCCTTAG
*evpQ*-*cyaA*-for	GC GGATCC ATAAAGATTGGGGAGGGGT
*evpQ*-*cyaA*-rev	GA AGATCT ATTTGGCTCAAGCAATGGT
*evpQ*-*cyaA*-check-rev	TTGCCGCAGATAGTCAAGCCGCT
P-*evpQ*_–__594 to__–__1_ for	CGGAATTCGAGTTATTAAACCCGTCAAG
P-*evpQ*_–__594 to__–__1_ rev	CGGGATCCTTTCGGCCTCACCTCTGATA
16S-qfor	ACTGAGACACGGTCCAGACTCCTAC
16S-qrev	TTAACGTTCACACCTTCCTCCCTA
*evpQ*-qfor	TGTGCTAATCGTCGGGGTTA
*evpQ*-qrev	TTCATTGCGCAGTTTACGGA

### Plasmid Construction

The pACYC*-evpQ-*HA is derived from pACYC-184 (Amersham), which carries the *evpQ*-HA gene under the control of tetracyclin resistance gene promoter. To construct, DNA fragment including the ribosome-binding site and complete ORF of *evpQ* were amplified by PCR from *E. piscicida* PPD130/91 genomic DNA using primer pair *evpQ*-com-for and *evpQ*-com-rev (Table 2). The PCR products, containing *Eco*RI and *Sca*I sites, were digested and ligated into pACYC-184 to generate pACYC-*evpQ-*HA. The *evpQ* gene together with its ribosome-binding site digested with *Bam*HI and *Bgl*II was inserted into pACYC-*escE*-*cyaA* (Lu et al., 2016), which was also digested with the same two restriction enzymes, acquiring pACYC-*evpQ*-*cyaA.* All the plasmids constructed were verified by DNA sequencing before being introduced into *E. piscicida* strains by electroporation. The strains obtained were verified by immunoblotting.

### Epitope Tagging of the Chromosomal Copy of *evpQ* With a 2HA Tag

To tag the chromosomal copy of *evpQ* with DNA encoding a double HA epitope, the λ Red recombination system was used as described (Datsenko and Wanner, 2000; Uzzau et al., 2001). Briefly, the forward primer (*evpQ*-2HA-for) containing the 3’-terminal sequence (without the stop codon) of *evpQ* followed by a sequence encoding a 2HA epitope and the reverse primer (*evpQ*-2HA-rev) corresponding to a chromosomal region downstream from *evpQ* were used to amplify the kanamycin (Km) resistance gene from pSU315. The PCR product was electroporated into competent cells of *E. piscicida* PPD130/91 wild-type strain, its isogenic mutant strain Δ*esrC*, Δ*rpoS*, or Δ*fur* strain, and each of which were transformed with pKD46 (Datsenko and Wanner, 2000). The colonies obtained were screened through probing with anti-HA antibody.

### Immunoblotting Analysis

Secreted proteins [extracellular proteins (E)] and bacterial lysates [total bacterial proteins (TBPs)] were prepared as described by Zheng and Leung (2007). ECPs and TBPs were loaded onto a NuPAGE 12% gel for electrophoresis in MES SDS running buffer (Invitrogen). Proteins on sodium dodecyl sulfate polyacrylamide gel electrophoresis (SDS-PAGE) gel were transferred onto polyvinylidene fluoride (PVDF) membrane (Millipore), before being probed with rabbit antibodies against HA (1:2,000) (Sigma), DnaK (1:10,000) (Cusabio Technology LLC), EseG (1:1,000) (Xie et al., 2010), EseE (1:1,000) (Yi et al., 2016), and EvpC (1:5,000) (Zheng and Leung, 2007).

### CyaA-Based Translocation Assay

CyaA translocation assay was conducted on EPC cells as previously described (Xie et al., 2015), and cyclic AMP (cAMP) levels were assayed by using an enzyme-linked immunoassay (ELISA) according to the instruction of the manufacturer (ARBOR ASSAYS).

### Mechanic Fractionation of EPC Cells

Infected EPC cells were fractionated as described by Coombes et al. (2007) with minor modifications. Briefly, EPC cells were seeded at 6 × 10^6^ cells per 100 mm diameter tissue culture dish 1 day before infection. Before infection, the *E. piscicida* culture reaching 0.5 at OD_540 nm_ were applied onto EPC cells, at an MOI of 15 in 3 ml M199 medium per dish. Dishes were maintained at 25°C in a 5% CO_2_ incubator for 30 min before the medium was aspirated, and DMEM supplemented with 16 μg/ml gentamycin was added. Infections were allowed to proceed for another 2 h before the EPC cells were harvested and resuspended in 300 μl homogenization buffer (HB) [250 mM sucrose, 3 mM imidazole, 0.5 mM EDTA (pH 7.4)] supplemented with halt protease inhibitor cocktail (Thermo Fisher Scientific). The cells were homogenized on ice by mechanical lysis using a 1.0-ml syringe with a 22-gauge needle. The lysate was spun for 15 min at 3,000 × *g* at 4°C. The supernatants were precipitated by methanol and chloroform as described by Wang et al. (2017) and dissolved in 1 × sodium dodecyl sulfate (SDS) loading buffer.

### Competitive Index in Blue Gourami Fish

Mixed competitive infection in naïve blue gourami (*Trichogaster trichopterus* Pallas) (9.54 ± 1.52 g) was performed to determine the contribution of EvpQ to pathogenesis. Overnight-cultured *E. piscicida* wild-type and Δ*evpQ*::*km* strains were subcultured at 1:40, respectively, and cultured at 25°C in TSB for 2.5 h. The bacteria were washed three times in PBS, and the OD_540_ was adjusted to 0.5. Equal amounts of bacteria were mixed and injected intramuscularly (i.m.) at 1 × 10^5^ CFU per fish. At 48 h post-inoculation, spleen and head kidney were dissected and homogenized, and a series dilution were spread onto TSA plates supplemented with colistin. The colonies obtained were patched onto TSA plate with colistin/kanamysin and TSA plate with colistin to determine the ratio of Δ*evpQ*::*km* strain to the wild-type strain. The competitive index (CI) value is the ratio of the Δ*evpQ*:*km* strain and wild-type strain within the output divided by their ratio within the input.

The experiments with fish were performed in strict accordance with the recommendations in the Guide for the Care and Use of Laboratory Animals of the Institute of Hydrobiology, Chinese Academy of Sciences.

### Statistical Analysis

Probability (*P*) values were calculated by two-way analysis of variance and least-significant difference (LSD) or Student’s *t-*test, as stated in the figure legends, and they were considered significantly different if the *P*-values were less than 0.05.

## Results

### EvpQ Is a Novel Secreted Protein

To understand the strategies that *Edwardsiella* spp. use to combat host immune system, our previous study searched new proteins secreted by T3SS through comparative proteomics on the extracellular proteins of the Δ*esaB* and Δ*esaB*Δ*esaN* strains. This approach is based on the fact that the *spiC* mutant over-secretes the *Salmonella* pathogenesis island-2 (SPI-2) effector proteins (Yu et al., 2004). *E. piscicida* EsaB is homologous with *Salmonella* SpiC (Liu et al., 2017). EsaN is an ATPase that energizes the transportation of T3SS effectors (Xie et al., 2010). The protein encoded by ORF02740 was found to be slightly hyper-secreted by Δ*esaB*Δ*esaN* strain than by Δ*esaB* strain (data not shown). This suggests that ORF02740 is secreted, not depending on T3SS. We named this protein as EvpQ (*Edwardsiella* virulent protein Q).

**FIGURE 1:**
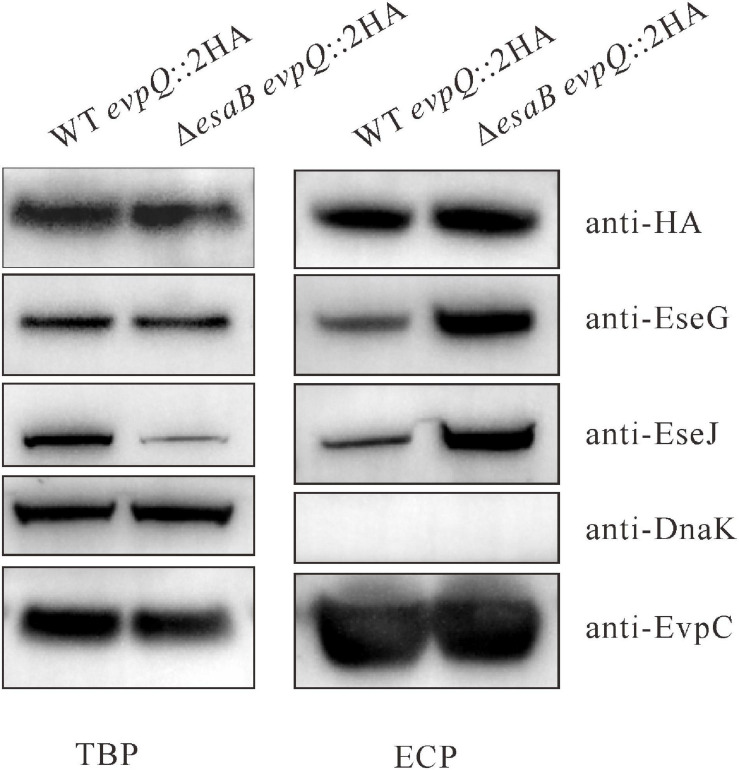
EvpQ is secreted by *Edwardsiella piscicida* in a manner different from T3SS effectors. Five percent of bacterial pellets (TBPs) and 10% of culture supernatants (ECPs) from similar amounts of WT *evpQ*:2HA and Δ*esaB evpQ*:2HA strains grown in Dulbecco’s modified Eagle’s medium (DMEM) were separated using sodium dodecyl sulfate polyacrylamide gel electrophoresis (SDS-PAGE) and transferred onto polyvinylidene fluoride (PVDF) membranes for probing with anti-HA (EvpQ), anti-EseG, anti-EseJ, anti-DnaK, and anti-EvpC.

To corroborate the secretion of EvpQ from the comparative proteomics, a 2HA tag was introduced at the C-terminal of *evpQ* on the genome of *E. piscicida* wild-type 130/91 and its isogenic Δ*esaB* strain. The secretion of EvpQ was probed with anti-HA antibody. EvpC, a major protein secreted via T6SS was used as a loading control (Zheng and Leung, 2007). As shown in Figure 1, dramatically increased secretion of T3SS effectors EseG and EseJ was detected from the Δ*esaB* strain as compared with the wild-type strain; however, no secretion difference can be discerned for EvpQ. Chaperone protein DnaK was not detected from the ECPs, indicating that the detection of EvpQ from the ECPs is not due to leakage from bacterial pellets (Figure 1). These results indicate that EvpQ is secreted in a manner different from T3SS effectors.

The *evpQ* gene encodes a peptide of 173-amino acid residues with a predicted molecular mass of 20.1 kDa, and a predicated isoelectric (pI) point of 9.3. Homologs of EvpQ were not detected from the other species of *Edwardsiella*, such as *Edwardsiella ictaluri* 93–146, *Edwardsiella anguillarum* ET080813, *Edwardsiella hoshinae* ATCC 35051, or *Edwardsiella tarda* KC-Pc-HB1. Using InterPro^1^ for informative analysis, the EvpQ protein was found to belong to peptidase_C70 super family. SWISS-MODEL analysis reveals that EvpQ shares 23.91% identity with type III effector protein AvrRpt2 from *Erwinia amylovora*. AvrRpt2 is a T3SS effector that manipulates the host to evade proteolysis (Mudgett and Staskawicz, 1999; Shindo and Van der Hoorn, 2008). The EvpQ protein was thereof speculated to be a novel virulence factor of *E. piscicida*.

### EvpQ Is Secreted at a T6SS-Dependent Manner

**FIGURE 2:**
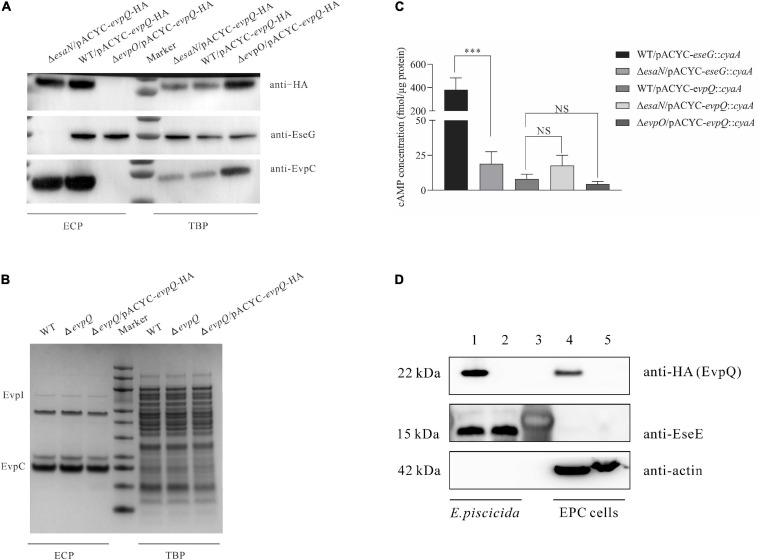
EvpQ is secreted at a T6SS-dependent manner and is translocated into eukayotic host. **(A)** EvpQ is secreted at a T6SS-dependent manner. TBPs and ECPs from each strain grown in DMEM were harvested at stationary phase, and equal amount of TBPs and ECPs were loaded for separating by SDS-PAGE and transferred onto PVDF membranes for immunoblotting. **(B)** Extracellular and intracellular profiles of *E. piscicida* strains. ECPs from similar amounts of *E. piscicida* strains grown in DMEM were separated using SDS-PAGE and stained with Coomassie blue. EvpI, EvpP, and EvpC are T6SS proteins secreted. **(C)** EvpQ does not depend on T3SS for its translocation. The EPC cells were infected with *E. piscicida* strains transformed with pACYC-*evpQ*::*cyaA*. At 2 h post-infection, the EPC monolayers were processed to examine the intracellular cAMP level as described in section “Materials and Methods.” Means ± SD from one representative experiment was shown. ^* **^P < 0.001; NS, not significant. **(D)** EvpQ is translocated into host cells. EPC cells were infected, respectively, with WT *evpQ*::2HA strain and Δ*evpQ* strain before fractionation by mechanical disruption. Cell lysates of EPC cells and bacteria were analyzed by immunoblotting using anti-HA, anti-EseE, and anti-actin antibodies. Lanes 1–2, *E. piscicida* cell lysates of WT *evpQ*::2HA strain and Δ*evpQ* strain, respectively; lane 3, protein marker; lanes 4–5, cell lysates of EPC cells infected, respectively, with WT *evpQ*::2HA strain and Δ*evpQ* strain.

T6SS plays a pivotal role in the pathogenesis of *E. piscicida* (Zheng and Leung, 2007). To investigate if EvpQ is secreted through T6SS, the pACYC-*evpQ-*HA was introduced into *E. piscicida* wild-type PPD130/91, T6SS-deficient Δ*evpO* strain, and T3SS-deficient Δ*esaN* strain. The expression of T3SS and T6SS was induced by culturing the aforementioned strains in DMEM. EvpO is an ATPase that energizes the transportation of T6SS substrates (Zheng and Leung, 2007). It was observed that the secretion of EvpQ was dependent totally on T6SS and not through T3SS (Figure 2A). The expression and secretion of T3SS effector EseG and T6SS substrate EvpC were examined as the control. As expected, EseG was not secreted from the Δ*esaN* strain and EvpC not from the Δ*evpO* strain, indicating that the strains are correct. EseG was not detected from the ECP of the Δ*esaN* strain, indicating that the detection of EvpQ from the Δ*esaN* strain is not due to its leakage from bacterial pellets (Figure 2A). Together, these results demonstrate that EvpQ depends on an active T6SS for its secretion.

Some regulators can also be secreted through bacterial secretion system, and deletion of regulator gene changes the extracellular protein profile (Rietsch et al., 2005). To learn whether EvpQ could be a secreted regulator, we examined the extracellular and intracellular protein profiles (ECPs and TBPs) of *E. piscicida* wild-type strain, Δ*evpQ* strain, and Δ*evpQ/*pACYC*-evpQ-*HA strain. As shown in Figure 2B, depletion or overexpression of EvpQ did not change the extracellular or intracellular protein profile of the *E. piscicida* strains. This result suggests that EvpQ is not a secreted regulator, and it could be a T6SS effector.

### EvpQ Is Translocated Into Host Cells

Will EvpQ be translocated into host cells? To answer this question, we constructed a reporter plasmid pACYC-*evpQ*::*cyaA*, expressing a chimeric protein EvpQ::CyaA, as described by Lu et al. (2016). When the CyaA fusion is translocated into the host cell cytoplasm, CyaA will convert ATP into cAMP in the presence of the eukaryotic-cell cytoplasmic protein calmodulin. The increase of cAMP is taken as the translocation of EvpQ protein. This method has been used in many pathogenic bacteria, including *E. piscicida*, to successfully monitor the translocation of bacterial T3SS effectors (Sory and Cornelis, 1994; Casper-Lindley et al., 2002; Xie et al., 2015). The pACYC-*evpQ*::*cyaA* was introduced into *E. piscicida* strains to infect EPC cells. At 2 h post-infection (hpi), the cAMP levels inside EPC cells were measured as a readout of translocation of EvpQ::*CyaA*. As shown in Figure 2C, the cAMP level in WT/pACYC-*eseG*::*cyaA*-infected cells was examined as the positive control (423.46 ± 70.12 fmol/μg protein), and in Δ*esaN*/pACYC-*eseG*::*cyaA*-infected cells as the negative control (18.86 ± 8.75 fmol/μg protein). It was found that the cAMP level in WT/pACYC-*evpQ::cyaA*-infected cells was 7.97 ± 3.59 fmol/μg protein, in Δ*esaN*/pACYC-*evpQ*::*cyaA*-infected cells was 17.65 ± 7.27 fmol/μg protein, and in Δ*evpO*/pACYC-*evpQ*::*cyaA*-infected cells was 4.45 ± 1.89 fmol/μg protein. No significant difference of cAMP levels was detected from the three strains examined. These data demonstrate that EvpQ is not translocated into host cells in a T3SS-dependent manner. However, we could not draw a conclusion that EvpQ cannot be translocated into host cells through T6SS. Considering that the cAMP system was primarily set up for T3SS effector identification (Sory and Cornelis, 1994), moreover, EvpQ is fused at the N-termini of CyaA (501 aa), this results in a large fusion protein, which could have blocked its translocation through T6SS tail tube.

Next, we labeled *evpQ* on the genome with 2HA tag (18 aa) using the λ Red recombinase method (Datsenko and Wanner, 2000). To localize EvpQ in infected host cells, EPC cells infected with the *E. piscicida* WT *evpQ::*2HA and Δ*evpQ* strains were subjected to mechanic fractionation. Actin was used as marker of host cell component in EPC cells. EseE, a T3SS chaperone of EseC (Yi et al., 2016), was used as a marker of bacterial components. EvpQ-2HA, but not EseE, was detected from the supernatants of the infected EPC cell lysates (cytosol and membrane) (Figure 2D). This indicates that this fraction was not contaminated by the bacteria. This data provide evidence on the translocation of EvpQ into EPC cells. Taken together, we have demonstrated that EvpQ is a T6SS effector that is translocated into eukaryotic host cells.

### EvpQ Facilitates the Replication of *E. piscicida* in Blue Gourami Infection Model

**FIGURE 3:**
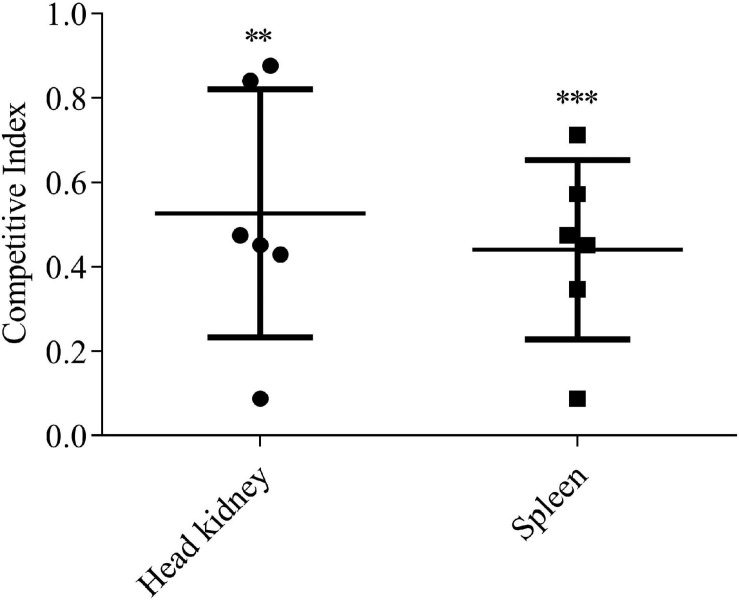
The Δ*evpQ* strain is less competitive than wild-type *E. piscicida*. Six naïve blue gourami fish were injected intramuscularly with a mixture of equal numbers of wild-type and Δ*evpQ*::*km* strains and killed 48 h post-injection. CIs from spleen and head kidney were given for individual fish, and means ± SD were shown by the horizontal lines. Student’s *t* test was used to calculate the *P-*value with the hypothetical mean of 1.0. ***P < 0.001; **P < 0.01.

The role of EvpQ in the pathogenesis of *E. piscicida* was investigated in blue gourami infection model by using CI assay. The equal number of the Δ*evpQ*:*km* strain and wild-type strain were mixed to inject blue gourami fish i.m. at 1 × 10^5^ CFU per fish. At 48 h post-inoculation, spleen and head kidney were dissected to determine the amount of Δ*evpQ*:*km* and wild-type strains. The CIs from head kidney were 0.53 ± 0.27, and the CIs from spleen were 0.44 ± 0.19 (Figure 3). As the CIs were significantly less than 1.0, we conclude that the deletion of *evpQ* significantly attenuates the virulence of *E. piscicida* PPD130/91.

### EvpQ Is Encoded by a Genomic Island, a Mobile Genetic Element

**FIGURE 4:**
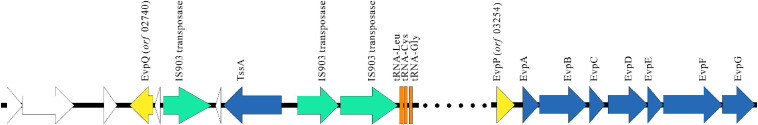
EvpQ is encoded by a genomic island far outsides T6SS gene cluster. Gene organization of T6SS gene cluster and the GI that encodes EvpQ. Data were from reference (Zheng and Leung, 2007) and our laboratory. Open reading frames with identifiable orthologs in the horizontal transferred fragment are labeled. Yellow arrows, T6SS effector genes; azure arrows, T6SS apparatus genes; green arrows, transposase genes; orange arrows, tRNA genes; white arrows, hypothetical genes.

With the help of MGE, many bacteria have accessorized their genomes with DNA from bacteria outside of their species or genus. The most common MGEs of horizontal transfer are genomic islands (GIs), plasmids, and bacteriophages. GIs are areas of the genome that are flanked by specific DNA sequences, which contain direct repeats and are often inserted in highly conserved genes, e.g., tRNA genes. The specific DNA sequences also carry genes coding for genetic mobility such as transposases (Jackson et al., 2011). The *evpQ* gene (*orf* 02740) is spaced by 513 genes from *evpP* (*orf* 03254), the first gene of the T6SS gene cluster. Three consecutive tRNA genes and three IS903 transposase genes were found upstream of *evpQ* (Figure 4). Moreover, by the DNAstar analysis, the mean GC content of this MGE that *evpQ* localizes is 49.0%, which is much lower than that of the genome of *E. piscicida* PPD130/91 (59.8%). This indicates that EvpQ is encoded by horizontal transferred genomic island. Besides, TssA was also encoded by the same the GI. TssA protein is reported to play a role in coordinating T6SS inner tube/sheath assembly (Dix et al., 2018). This indicates that the genomic island is probably a pathogenicity island.

### Transcription of *evpQ* Is Positively Regulated by EsrC and Negatively by Fur

**FIGURE 5:**
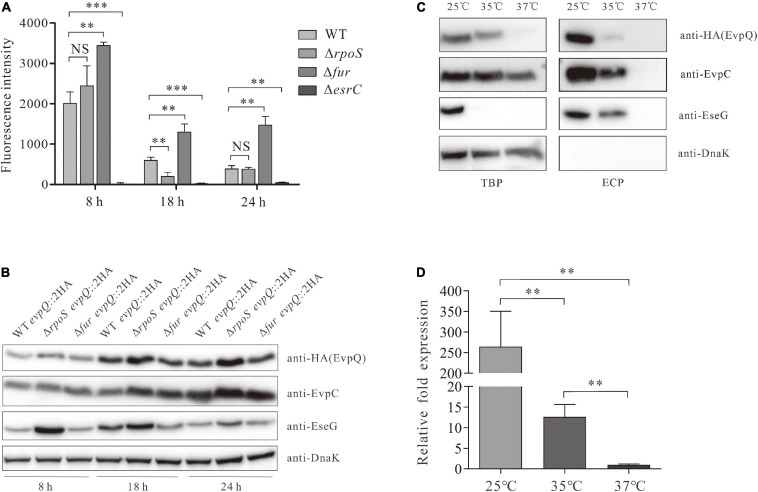
EvpQ is under the control of EsrC, RpoS, Fur, and temperature. **(A)** Comparison of the transcriptional level of *evpQ* in WT, Δ*esrC*, Δ*rpoS*, and Δ*fur* strains. The DNA fragment (bp -594 to -1) upstream of *evpQ* was inserted into the promoterless *gfp* shuttle vector pFPV25 to create pFPV-*evpQ*_–__594 to__–__1_. This plasmid was then introduced into *E. piscicida* strains. At 8, 18, and 24 hps in DMEM, the fluorescence intensity of those *E. piscicida* strains were evaluated by using a microplate reader. Means ± SD from one representative experiment was shown. ***P < 0.001; **P < 0.01; NS, not significant. **(B)** Examination on the steady-state protein level of EvpQ in different *E. piscicida* strains at 8-, 18-, and 24-h post-subculture in DMEM. This experiment was repeated separately for at least three times, and one representative blot was shown. **(C)** The expression and secretion of EvpQ from *E. piscicida* wild type cultured in DMEM at different temperatures. ECPs and TCPs from similar amounts of the WT *evpQ*:2HA cultured at 25, 35, or 37°C were probed with anti-HA, anti-EvpC, anti-EseG, and anti-DanK antibodies. **(D)** The transcription levels of *evpQ* from *E. piscicida* wild type cultured in DMEM at different temperatures. The mRNA levels of *evpQ* from the wild-type strain cultured at 25, 35, or 37°C were examined by qRT-PCR. 16S rRNA was used as the reference gene. Transcription levels of *evpQ* relative to that of 16S rRNA are presented (i.e., relative fold changes in gene expression). Data are presented as means ± SD. **P < 0.01.

RpoS inhibits *E. piscicida* T6SS by blocking RpoD-mediated transcription of *esrB* (Yin et al., 2018). EsrB positively regulates T6SS through EsrC (Tan et al., 2005; Zheng et al., 2005; Chakraborty et al., 2011). EsrC and Fur compete to bind directly to the Fur box in the promoter of *evpP* (Chakraborty et al., 2011). To learn whether these proteins also regulate the transcription of the novel T6SS effector EvpQ, a DNA fragment (bp -594 to -1) upstream of *evpQ* was inserted into the promoterless *gfp* shuttle vector pFPV25 to create pFPV-594 to -1. The resulting construct (pFPV-594 to -1) was introduced into *E. piscicida* WT, Δ*rpoS*, Δ*fur*, Δ*esrB*, and Δ*esrC* strain, respectively. At 8-, 18-, and 24-h post-subculture (hps) in DMEM, the activity of this putative promoter in the aforementioned strains were tested with a microplate reader (Synergy neo2, Biotek, United States). As seen in Figure 5A, similar levels of fluorescent intensity were detected from the Δ*rpoS* strain and wild-type strain, whereas dramatically elevated fluorescence intensity was detected from the Δ*fur* strain, and sharply decreased levels of fluorescence intensity were detected in the Δ*esrC* strain at each time point examined (8, 18, and 24 hps). These data indicate that when culturing in DMEM, *evpQ* is negatively regulated by Fur and positively regulated by EsrC; however, RpoS seems not to be involved in the transcription of *evpQ.*

### The Steady-State Protein Level of EvpQ Is Negatively Controlled by RpoS

The steady-state protein level of EvpQ in the aforementioned strains was examined. As shown in Figure 5B, similar levels of steady-state EvpQ were detected from WT, Δ*rpoS*, and Δ*fur* strain at 8 hps. At 18 or 24 hps, the steady-state protein level of EvpQ increased slightly in WT or Δ*fur* strain, meanwhile it increased starkly in Δ*rpoS* strain and reached maximum at 24 hps. As a control, protein level of type III effector EseG was examined. It was found that EseG was also negatively controlled by RpoS. The highest protein level of EseG was detected in Δ*rpoS* strain at 8 hps, and it deceases with its growth. Equal amounts of proteins were loaded per lane as indicated by the DnaK protein level. These data indicate that both EvpQ and EseG are negatively controlled by RpoS, and intracellular EvpQ increases while EseG decreases with its growth.

### The Expression and Secretion of EvpQ Are Influenced by Temperature

*Edwardsiella piscicida* PhoQ sensor domain detects temperatures through a conformational change of its secondary structure, and it regulates the types III and VI secretion systems through direct activation of *esrB* (Chakraborty et al., 2010). *E. piscicida* types III and VI secretion systems are activated from 23 to 35°C, but they are suppressed at or below 20°C, at or above 37°C. Will the protein level of EvpQ be subject to temperature? To answer this question, we compared the intracellular and extracellular protein levels of EvpQ at 25, 35, and 37°C from the WT *evpQ*::2HA strain cultured in DMEM. As shown in Figure 5C, the expression and secretion of EvpQ decreased sharply at 35°C as compared with that at 25°C, and EvpQ almost failed to be detected from TBP and ECP when WT *evpQ*::2HA strain was cultured at 37°C. Meanwhile, the secretion level of T3SS effector EseG mildly decreased at 35°C as compared with that at 25°C. This indicates that T6SS effector EvpQ is more susceptible to temperature change than T3SS effector EseG.

To investigate whether the decreased steady-state protein level of EvpQ resides in the decreased transcription level of *evpQ* when the culture temperature increases. We compared the transcript levels of *evpQ* gene from the wild-type strain cultured at 25, 35, and 37°C, respectively. 16S rRNA was used as the reference gene. The transcript levels of *evpQ* were significantly (*P* < 0.01) downregulated by 265- and 21-fold, respectively, at 37 and 35°C when comparing with that at 25°C, and downregulated by 13-fold when comparing between 37 and 35°C (Figure 5D). Thus, the deceased expression level of EvpQ is due to the decreased transcription of *evpQ* with the elevation of culture temperature.

## Discussion

T6SS is a versatile machinery playing roles in both pathogenesis and interbacterial competition by translocating T6SS effectors. T6SS effectors are loaded into the inner tube or associate with the spike trimer during T6SS assembly (Silverman et al., 2013; Flaugnatti et al., 2016; Hernandez et al., 2020). Contraction of the sheath propels the inner tube/spike, which allows perforation of the target cell membrane, delivering effectors into the cytosol of target cells (Journet and Cascales, 2016). Sory and Cornelis (1994) set up a system to examine the translocation of T3SS effector. By this method, effector candidate was fused at the N-terminal of CyaA, and CyaA converts ATP into cAMP in the presence of calmodulin when the fusion protein is translocated into host cell cytoplasm. The increase in cAMP of taken as the translocation of T3SS effector candidate. In this study, EvpQ was fused at the N-terminal of CyaA (501 aa), while we failed to detect the translocation of EvpQ::CyaA into EPC cells. It is speculated that this method may not be applicable to indicate the translocation of T6SS effector. On one aspect, the large size of the fusion protein may block its translocation through T6SS inner tube; on the other aspect, the fusion of CyaA to EvpQ could have changed the ability of EvpQ to attach to spike trimer, thereof disabling its translocation. When 2HA tag (18 aa) was fused at the C-terminal of *evpQ* on the genome, the translocation of EvpQ::2HA (191 aa) was successfully detected by mechanic fractionation of EPC cells infected. EvpQ::2HA could have been translocated through the T6SS tail tube.

EsrB elaborately controls T6SS expression through EsrC (Zheng et al., 2005). Fur requires iron binding for its activity, and the activated Fur binds to promoters of iron-responsive genes to repress their transcription under iron replete conditions (Escolar et al., 1999). Fur was reported to represses *Salmonella* SPI-2 expression that is activated inside macrophages and under acidic conditions (Choi et al., 2014). In *E. piscicida*, Fur senses high iron concentration and binds directly to the Fur box in the promoter of *evpP* to inhibit EsrC’s binding to the same region (Chakraborty et al., 2011). EvpQ was negatively regulated by Fur and positively regulated by EsrC. Whether Fur and EsrC compete in binding with the promoter of EvpQ awaits further study. Moreover, we revealed in this study that the steady-state protein level of EvpQ was negatively controlled by RpoS when *E. piscicida* was cultured in DMEM. Accordingly, Yin et al. (2018) revealed that ETAE_2037 of *E. piscicida* EIB202 (renamed as EvpQ in the current study) was negatively regulated by the sigma factor RpoS when cultured in DMEM, as revealed by RNAseq. Our study failed to detect the difference in the transcription of *evpQ* between wild-type and Δ*rpoS* strains at 8 and 24 hps. This is probably due to the low sensibility of fluorescence detection system, when comparing with the RNAseq assay.

**FIGURE 6:**
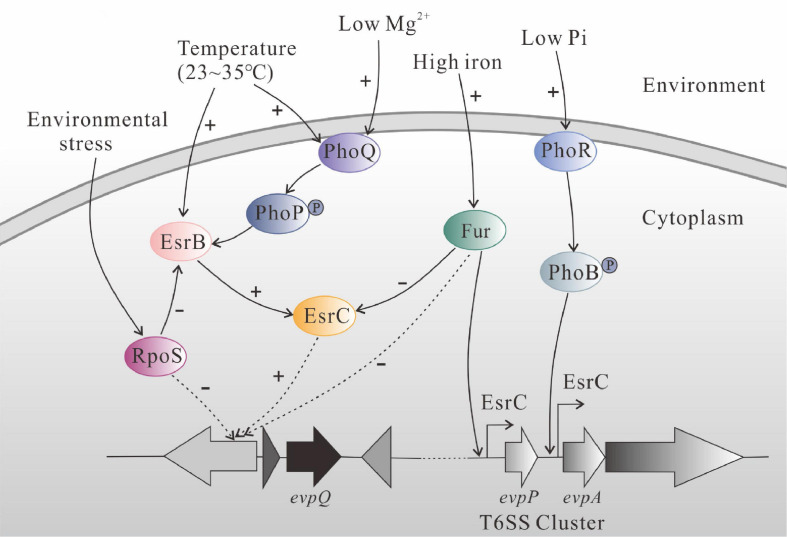
Schematic diagram of regulatory network of EsrC, RpoS, and Fur on EvpQ and T6SS gene cluster in *E. piscicida* PPD130/91, based on reference papers and our results from the current study. PhoQ senses the change of ambient temperature (23∼35°C) and transmits the signal to PhoP, and the phosphorylated PhoP binds directly to the PhoP box within the promoter region of *esrB* to activate its transcription (Chakraborty et al., 2010). The activated EsrB protein upregulates the transcription of T6SS through EsrC (Zheng et al., 2005; Chakraborty et al., 2010). High concentration of iron activates the Fur protein, and activated Fur binds directly to the Fur box in the promoter of T6SS effector gene *evpP*. The binding of Fur inhibits the binding of EsrC to the same region (Chakraborty et al., 2011). The EvpQ encoded by the genomic island is also negatively regulated by Fur and positively regulated by EsrC, while it remains to be resolved whether EsrC and Fur directly bind the promoter of *evpQ*. The sigma factor RpoS, antagonizing the expression of *esrB* (Yin et al., 2018), negatively controls the expression of EvpQ.

Based on references and our results, we drew a schematic diagram to summarize regulatory role of EsrC, RpoS, and Fur on EvpQ and T6SS gene cluster (Figure 6). PhoQ senses the change of the ambient temperature (23∼35°C) and transmits the signal to PhoP, and the phosphorylated PhoP binds directly to the PhoP box within the promoter region of *esrB* to activate its transcription (Chakraborty et al., 2010). The activated EsrB protein upregulates the transcription of T6SS through EsrC (Zheng et al., 2005: Chakraborty et al., 2010). High concentration of iron activates the Fur protein, the activated Fur binds directly to the Fur box in the promoter of T6SS effector gene *evpP*, and the binding of Fur inhibits the binding of EsrC to the same region (Chakraborty et al., 2011). The novel T6SS effector *evpQ* that is localized on the genomic island is also negatively regulated by Fur and positively regulated by EsrC; it remains to be resolved in the near future whether or not EsrC and Fur binds the promoter of *evpQ* directly. RpoS can block RpoD-mediated transcription of *esrB*, antagonizing the expression of *esrB*, thereof inhibiting the expression of *E. piscicida* T6SS proteins (Yin et al., 2018). RpoS negatively controls the expression of EvpQ. RpoS represses promoters containing a -6G in their respective discriminator sequences (Yin et al., 2018). Whether or not RpoS represses EvpQ through binding to a -6G in the promoter of *evpQ* awaits further study.

EvpQ is encoded by a genomic island, and it exists only in *E. piscicida* but not in any other species of the genus *Edwardsiella*, such as *E. ictaluri*, *E. anguillarum*, *E. hoshinae*, or *E. tarda.* Alike EvpQ, GtgE localizes on the Gifsy-2 prophage of *Salmonella* Typhimurium (Ho et al., 2002). *S.* Typhimurium can infect a broad range of vertebrate species, whereas *Salmonella* Typhi only infects humans. GtgE is not encoded by *S.* Typhi, and introducing GtgE into *S.* Typhi enables this human-adapted serovar to survive within non-permissive host cells (Spanò and Galán, 2012). *E. ictaluri* only infects fish in the *siluriformes*, while *E. piscicida* infects fish from wide orders. It is interesting to investigate in the future whether introducing EvpQ into *E. ictaluri* extends its hosts or not.

EvpQ shares similarity with papain-like cysteine protease AvrRpt2, which suppresses plant immunity by modulating auxin signaling and cleavage of the membrane localized defense regulator RIN4 (Axtell et al., 2003; Mackey et al., 2003; Cui et al., 2013). AvrRpt2 also specifically suppresses pathogen-associated molecular pattern (PAMP)-induced activation of MPK4 and MPK11 but not of MPK3 and MPK6 (Eschen-Lippold et al., 2016). Whether EvpQ manipulate and fine-tune MPK signaling pathway upon its translocation into fish hosts awaits further investigation.

## Data Availability Statement

The original contributions presented in the study are included in the article/supplementary material, further inquiries can be directed to the corresponding author.

## Ethics Statement

The animal study was reviewed and approved by the Animal Ethical and Welfare Committee, Institute of Hydrobiology, Chinese Academy of Sciences.

## Author Contributions

DL, YL, XL, TH, and SS performed the experiments and prepared materials for the manuscript. HX and PN designed and supervised the experiments, and interpreted the data. DL, YL, and HX wrote the manuscript. All authors contributed to the article and approved the submitted version.

## Conflict of Interest

The authors declare that the research was conducted in the absence of any commercial or financial relationships that could be construed as a potential conflict of interest.

## Footnotes

1 http://www.ebi.ac.uk/interpro/scan.html
